# Gut microbiota composition in patients with advanced malignancies experiencing immune-related adverse events

**DOI:** 10.3389/fimmu.2023.1109281

**Published:** 2023-02-20

**Authors:** Xinyu Liu, Hao Tang, Qingyang Zhou, Yanlin Zeng, Bo Lu, Dan Chen, Yue Li, Jiaming Qian, Minjiang Chen, Jing Zhao, Yan Xu, Mengzhao Wang, Bei Tan

**Affiliations:** ^1^ Department of Gastroenterology, Peking Union Medical College Hospital, Peking Union Medical College & Chinese Academy of Medical Science, Beijing, China; ^2^ Eight-year Medical Doctor Program, Peking Union Medical College & Chinese Academy of Medical Science, Beijing, China; ^3^ Department of Internal Medicine, Peking Union Medical College Hospital, Peking Union Medical College & Chinese Academy of Medical Science, Beijing, China; ^4^ School of Medicine, Tsinghua University, Beijing, China; ^5^ Department of Gastroenterology, Beijing Hospital, National Center of Gerontology, Institute of Geriatric Medicine, Chinese Academy of Medical Science, Beijing, China; ^6^ Department of Respiratory and Critical Care Medicine, Peking Union Medical College Hospital, Peking Union Medical College & Chinese Academy of Medical Science, Beijing, China

**Keywords:** gut microbiome, immune-related adverse events, immune-related colitis, metabolic pathways, anti-PD-1 therapy

## Abstract

**Introduction:**

The gut microbiota is implicated in the occurrence and severity of immune-related adverse events (irAEs), but the role it plays as well as its causal relationship with irAEs has yet to be established.

**Methods:**

From May 2020 to August 2021, 93 fecal samples were prospectively collected from 37 patients with advanced thoracic cancers treated with anti-PD-1 therapy, and 61 samples were collected from 33 patients with various cancers developing different irAEs. 16S rDNA amplicon sequencing was performed. Antibiotic-treated mice underwent fecal microbiota transplantation (FMT) with samples from patients with and without colitic irAEs.

**Results:**

Microbiota composition was significantly different in patients with and without irAEs (P=0.001) and with and without colitic-type irAEs (*P*=0.003). *Bifidobacterium*, *Faecalibacterium*, and *Agathobacter* were less abundant and *Erysipelatoclostridium* more abundant in irAE patients, while *Bacteroides* and *Bifidobacterium* were less abundant and *Enterococcus* more abundant in colitis-type irAE patients. Major butyrate-producing bacteria were also less abundant in patients with irAEs than those without (P=0.007) and in colitic vs. non-colitic irAE patients (*P*=0.018). An irAE prediction model had an AUC of 86.4% in training and 91.7% in testing. Immune-related colitis was more common in colitic-irAE-FMT (3/9) than non-irAE-FMT mice (0/9).

**Conclusions:**

The gut microbiota is important in dictating irAE occurrence and type, especially for immune-related colitis, possibly by modulating metabolic pathways.

## 1 Introduction

Immune checkpoint inhibitors (ICIs) have become the “fourth pillar” of cancer management. ICIs have improved survival outcomes for patients with various types of cancer, including advanced lung cancer (1), which is the leading cause of cancer death worldwide (2). However, some patients taking immunotherapy develop immune-related adverse events (irAEs), ultimately limiting the full clinical application and potential of immunotherapy (3, 4). IrAEs represent a special class of toxicity caused by immune system overactivation induced by ICIs, leading to their temporary or permanent discontinuation and consequent life-threating tumor progression. There are still no sufficiently specific nor sensitive biomarkers to predict irAE occurrence.

Recently, the gut microbiota has been shown to play a role in shaping ICI efficacy. Baseline microbiota composition has been reported to be different in responders and non-responders to ICIs in various cancers, and fecal microbiota transplantation (FMT) from ICI responders improves ICI efficacy both preclinically (5–7) and clinically (8, 9). A recent clinical trial reported increased response rates to ICIs in metastatic renal carcinoma patients after taking bifidogenic probiotics (10). The microbiota may also influence irAE occurrence, and a few studies have now explored baseline gut microbiota differences in patients with and without colitic irAEs. Indeed, some specific intestinal bacteria appear to distinguish patients without or with mild irAEs from those with severe irAEs (11–15). Moreover, Wang et al. reported two cases of refractory colitic irAE successfully treated with FMT from healthy donors (16). However, most of these studies remain limited to the study of colitic irAEs in melanoma patients.

To broaden our knowledge of the relationship between the gut microbiota, ICI response, and irAE occurrence, here we studied the gut microbiota of patients experiencing a spectrum of irAEs in different cancers, especially lung cancer. We first assessed changes in microbiota composition before and after anti-PD-1 therapy and irAE treatment. Then, we analyzed differences in microbiota composition in patients: (i) with and without irAEs; (ii) with irAEs of different severity; and (iii) with colitic and non-colitic irAEs. To explore the underlying mechanisms, we investigated differences in predicted microbiota function and butyrate production in different patient subgroups. Furthermore, we performed patient-to-mouse FMT experiments to explore the causal relationship between microbiota composition and immune-related colitis.

## 2 Methods

### 2.1 Patient enrollment

Fifty patients were consecutively enrolled into the anti-PD-1-treated lung cancer cohort between May 2020 and August 2021 at the Lung Cancer Center, Peking Union Medical College Hospital, Beijing, China according to the following inclusion criteria: (i) aged 18-75 years; (ii) advanced thoracic cancers; and (iii) initially treated with anti-PD-1 therapy. Thirteen patients were excluded due to unblinding of placebo recipients or for other reasons. Patients with irAEs were simultaneously consecutively enrolled according to the inclusion criteria: (i) aged 18-75 years; (ii) diagnosed with an advanced malignant tumor; and (iii) developed irAEs after anti-PD-1 therapy. For both groups, exclusion criteria were: (i) patients with unstable vital signs; and (ii) patients exposed to antibiotics and/or probiotics within four weeks of enrolment.

The Ethical Committee of Peking Union Medical College Hospital approved the study protocol (No. ZS-3037). Written informed consent was obtained from all patients. This clinical study was registered on the Chinese Clinical Trial Register (ChiCTR-2000032088).

### 2.2 Clinical and demographic information collection

Demographic and clinical information including gender, age, tumor type, tumor stage, previous treatment, evaluation of treatment efficacy, progression-free survival (PFS), irAE type, and irAE grade were collected. Treatment efficacy was assessed according to RECIST version 1.1 criteria (17). PFS was defined as time from initiation of ICI therapy to first clinical and/or radiographically confirmed progression. IrAEs were monitored from the initiation of ICI therapy to August 31, 2021. IrAEs were diagnosed according to National Comprehensive Cancer Network (NCCN) guidelines on ICI-related toxicities without restrictions as to the involved organs (18). All clinical data were collected by experienced physicians.

### 2.3 Intestinal microbiota analysis

For patients started on anti-PD-1 therapy, fresh fecal samples were prospectively collected before and after ICI therapy. For patients with irAEs, fresh fecal samples were collected before irAE treatment, after irAE treatment, and after resuming ICIs. All fecal samples were stored in the Clinical Biobank, Medical Research Center, Peking Union Medical College Hospital, Chinese Academy of Medical Sciences.

Fecal samples were subjected to microbiota analysis by 16S rDNA amplicon sequencing on the Illumina MiSeq (PE300) sequencing platform (Illumina, San Diego, CA) (19). Operational taxonomic units (OTUs) were detected with QIIME2 (20) and grouped according to phylum and genus. Differences in microbiota composition were compared according to relative abundance of these two levels. α-diversity was assessed according to the observed species, Shannon index, and Chao1 index, while β-diversity was assessed by Bray-Curtis and weighted UniFrac distances. Bray-Curtis distances were also used for ordination by principal coordinate analysis (PCoA), and differences in composition structure were assessed by Adonis and analysis of molecular variance (AMOVA) (21). The multiple response permutation procedure (MRPP) (22) was based on OTUs. Species with statistically significant differences between groups were evaluated by linear discriminant analysis effect size (LEfSe) (23). Functional prediction of microbiota differences was performed using Tax4fun (24) and STAMP (Statistical Analysis of Metagenomic Profiles) (25) using functional inferences from the Kyoto Encyclopedia of Gene and Genomes (KEGG) database. The abundances of the main butyrate-producing bacteria (*Faecalibacterium*, *Agathobacter*, *Roseburia*, *Subdoligranulum*, *Ruminococcus_gnavus_group*, *Megasphaera*, *Phascolarctobacterium*, *Flavonifractor*, *Eubacterium_ruminantium_group*, *Coprococcus*, *Eubacterium_hallii_group*, *Oscillibacter*, *Butyricicoccus*, *Butyricimonas*, *Anaerostipes*, *Odoribacter*, *Porphyromonas*, *Eubacterium_ventriosum_group*, *Oscillospira*, and *Butyrivibrio*) were compared.

### 2.4 FMT experiments in mice

#### 2.4.1 Mice and interventions

Six- to 8-week-old male C57BL/6 mice were fed under specific pathogen–free conditions. Mice were treated with a cocktail of antibiotics (ANVM: 4 mg/mL ampicillin, 2 mg/mL neomycin, 4 mg/mL metronidazole, 2 mg/mL vancomycin) 250 μL each day by oral gavage. 18 mice were randomly divided into two groups, colitic-irAE-FMT and non-irAE-FMT, receiving FMT from three patients with colitic irAE and two patients without irAE after anti-PD-1 therapy, respectively. The antibiotic pre-treated mice were first gavaged with 150 μL of fecal suspension every other day for 14 days, then 250 μg anti-PD-1 and 100 μg anti-CTLA-4 monoclonal antibodies were injected intraperitoneally, with FMT continued every other day for 10 days. All mice were euthanized at the last day of injection or on the verge of death. The Experimental Animal Ethics Committee of Peking Union Medical College Hospital approved the study protocol (XHDW-2022-066).

#### 2.4.2 Colitis evaluation and dynamic microbiota analysis

Body weight and colon length were recorded, and the disease activity index (DAI) (26) and colitis scores (by histopathological analysis) (27) were evaluated by an independent investigator.

Slides were incubated with primary antibodies targeting CD4 (#70437, Leica Biosystems, Wetzlar, Germany), CD8 (#70349, Leica Biosystems), and CD20 (#71902, Leica Biosystems) at 4°C overnight and then incubated with Polymer Detection System reagents (PV-9000, ZSGB BIO, Beijing, China), visualized with DAB (ZLI-9019, ZSGB BIO), and counterstained with hematoxylin. Positively stained cells were counted in ten randomized fields (40× objective) under a light microscope by two experienced pathologists. Immunohistochemistry (IHC) scores were calculated as the mean positive cell percentage and degree of staining.

Colon tissue was treated with TRIzol reagent (Invitrogen, Waltham, MA). Extracted RNA was then reverse transcribed into cDNA using the Maxima First Strand cDNA Synthesis Kit (Thermo Fisher Scientific, Waltham, MA). Quantitative real-time polymerase chain reaction (qRT-PCR) was performed using the S1000 PCR thermocycler (BioRad, Hercules, CA). The results were analyzed using Alpha Innotech 2000 software (ProteinSimple, Santa Clara, CA) and presented as the ratio of the relative absorbance of glyceraldehyde-3-phosphate dehydrogenase (*Gapdh*) as a housekeeping gene using the 2^-ΔΔCT^ method. Primer sequences are presented in **Supplementary Table 1**.

Feces of three representative mice in each group were collected at baseline, after antibiotic treatment, after FMT, and before euthanasia. The samples were also subjected to microbiota analysis by 16S rDNA amplicon sequencing as previously described, while amplicon sequence variants (ASVs) were detected with QIIME2 (20).

### 2.5 Statistical analyses

Statistical analysis was performed using R software v4.0.3 and GraphPad Prism v8.3.1 (GraphPad Software, La Jolla, CA). Continuous variables are expressed as means ± SD or medians and interquartile ranges (IQR), as appropriate. Categorical variables are expressed as numbers and percentages or frequencies. Demographic and clinical characteristics were compared with Pearson’s chi-squared test, Fisher’s exact test, or the Wilcoxon rank-sum test between two groups as appropriate. Comparisons of bacterial abundance, α-diversity, and β-diversity were performed with *t*-tests or Wilcoxon rank-sum tests between two groups, as appropriate. Survival was estimated by the Kaplan–Meier method, and differences in survival were evaluated with the log-rank test. Random forest (RF) analysis was performed based on genus abundance. The analytic data were randomly sampled as an 80/20 split into the training (80%) and test (20%) sets. We selected different numbers of genera to build a random forest (RF) model, screened the main genera by MeanDecreaseAccuracy and MeanDecreaseGin (28), cross-validated the model, and drew a receiver operating characteristic (ROC) curve to evaluate the optimal model describing the main differential genera between groups. A two-sided *P* < 0.05 was considered statistically significant.

## 3 Results

### 3.1 Patient demographics and clinical characteristics

**Figure 1:**
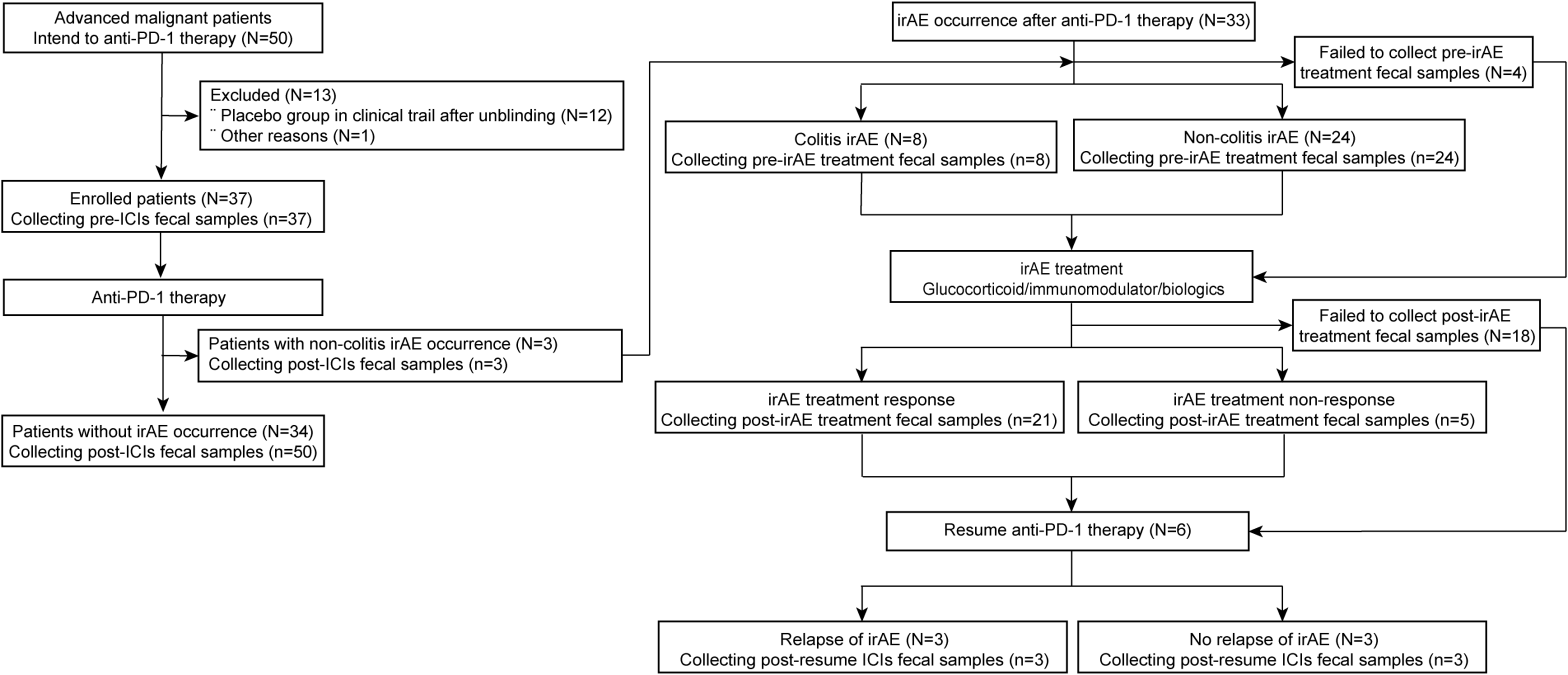
Flow diagram of participant enrollment and fecal sample collection in the anti-PD-1 therapy and irAE cohorts. N, number of patients; n, number of samples. PD-1, programmed death-1; irAE, immune-related adverse event; ICIs, immune checkpoint inhibitors.

Among the 37 patients taking anti-PD-1 therapy, three patients developed irAEs. An additional 33 patients with irAEs were included, four without pre-irAE treatment fecal samples. Thus, a total of 34 patients without irAEs (from patients with thoracic cancers taking anti-PD-1 therapies) and 32 patients with irAEs (from both cohorts) were included in the microbiota analysis (**Figure 1** and **Supplementary Figure S1**).

Among patients without irAEs, 47.1% and 41.2% had histologically confirmed adenocarcinoma and squamous cell carcinoma of the lung, respectively. 97.1% of patients were treated with anti-PD-1 therapy combined with chemotherapy or targeted therapy. Anti-PD-1 therapy resulted in partial responses (PR) in 52.9% and stable disease (SD) in 41.2% of patients, with a median PFS of 160 days. During subsequent anti-PD-1 therapy, four patients progressed: one patient from PR to SD, two patients from SD to progressive disease (PD), and one patient from PR to PD (**Supplementary Table 2** and **Supplementary Figure S1**).

Among patients with irAEs, 78.1% had lung cancer and 90.6% were treated with anti-PD-1 therapy combined with chemotherapy or targeted therapy. In this group, anti-PD-1 therapy was also mainly effective; PR in 56.3% and SD in 40.6%, with only one patient developing PD. The median PFS was 249.5 days. Grade 1-2 and grade 3-4 irAEs were present in 40.6% and 59.4% of patients, respectively, with 84.4% having one irAE and 15.6% multiple irAEs. There were 38 irAEs in total: colitis (8/38), pneumonitis (7/38), asymptomatic elevations in amylase/lipase or pancreatitis (6/38), transaminitis (5/38), rashes (4/38), myocarditis (3/38), acute kidney injury (3/38), myositis (1/38), and hyperglycemia-related diabetic ketoacidosis (1/38) (**Supplementary Table 2**).

After treatment with glucocorticoids, immunomodulators, or biologics, 21 samples were obtained from patients achieving irAE remission and 5 samples from patients who failed to respond. Among irAE remission patients, 6 patients resumed anti-PD-1 therapy but 3 relapsed with further irAEs (**Supplementary Table 2**; **Figure 1** and **Supplementary Figure S1**).

### 3.2 Differences in the gut microbiota of patients with and without irAEs

**Figure 2:**
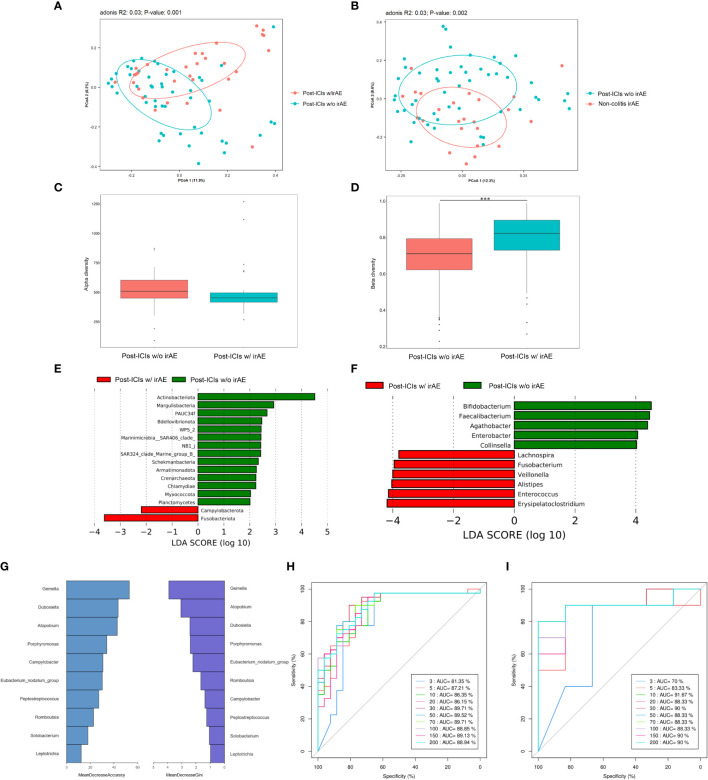
The microbiota of patients with and without irAEs. **(A)** PCoA plot of intestinal microbiota in patients with and without irAEs; **(B)** PCoA plot of intestinal microbiota in patients with non-colitic irAEs and patients without irAEs; **(C)** Box plot of α-diversity by observed species; **(D)** Box plot of β-diversity by Bray-Curtis distance of intestinal microbiota; LEfSe analysis of intestinal microbiota at the phylum level **(E)** and genus level **(F)**; Mean decrease in accuracy and mean decrease in Gini coefficient **(G)**, training set AUC **(H)**, and test set AUC **(I)** by random forest analysis at the genus level. AUC, area under the curve; ICIs, immune-checkpoint inhibitors; irAE, immune-related adverse event; LEfSe, LDA effect size; PCoA, principal coordinates analysis; w/, with; w/o, without. ***P < 0.001.

We compared differences in microbiota composition between patients who did and did not develop irAEs at the timepoint of sample collection after anti-PD-1 therapy. Fifty samples from 34 patients without irAEs and 32 samples from 32 patients with irAEs were finally included in the analyses. The demographic and clinical characteristics of these patients were generally balanced between the two groups (**Supplementary Table 2**). There were 1664 common OTUs and 1638 and 1539 differential OTUs in patients who had taken ICIs and did and did not develop irAEs, respectively. A PCoA plot revealed significant differences in microbiota composition between patients who did and did not develop irAEs (Adonis: *P*=0.001) (**Figure 2A**). These differences in microbiota structure were also confirmed by MRPP (*P*=0.001) and AMOVA (*P*<0.001) analyses. These differences persisted when patients with colitic irAEs were excluded from the analysis (Adonis: *P*=0.002) (**Figure 2B**). The median α-diversity in the non-irAE group was higher than that of the irAE group, with a trend toward a significant difference in observed species (*P*=0.06) (**Figure 2C**). There were also differences in β-diversity as assessed by Bray-Curtis and weighted Unifrac distances (*P*<0.001) (**Figure 2D**).

LEfSe analysis revealed that patients who developed irAEs had a higher abundance of *Fusobacteriota* and a lower abundance of *Actinobacteriota* at the phylum level and a higher abundance of *Erysipelatoclostridium* and lower abundance of *Bifidobacterium*, *Faecalibacterium*, and *Agathobacter* at the genus level compared with those not developing irAEs (**Figures 2E, F**).

Finally, RF analysis established a predictive model for irAE occurrence with AUCs of 86.35% in the training set and 91.67% in the test set consisting of ten main differential genera led by *Gemella*, *Dubosiella*, and *Atopobium* (**Figures 2G–I**).

We further compared the 13 patients with grade 1-2 irAEs and 19 patients with grade 3-4 irAEs. There were no significant differences in demographic and clinical characteristics between groups, except for the gender (*P*=0.029) (**Supplementary Table 3**). There were 1122 common OTUs and 180 and 2000 differential OTUs in grade 1/2 irAE and grade 3/4 irAE patients, respectively (**Supplementary Figure S2A**). Histograms showed that the microbiota composition was different between grade 1/2 irAE and grade 3/4 irAE patients at the phylum and genus levels (**Supplementary Figures S2B, C**), as did the PCoA plot (Adonis: *P*=0.068; MRPP: *P*=0.058; AMOVA: *P*=0.065). The microbiota composition of grade 3/4 irAE patients was more dispersed (**Supplementary Figure S2D**), with the β-diversity calculated by Bray-Curtis and weighted Unifrac distances supporting these differences (*P*<0.001) (**Supplementary Figure S2E**). However, there was no significant difference in α-diversity between these two groups (*P*=0.69). LEfSe analysis revealed that grade 3/4 irAE patients had a higher abundance of *Streptococcus* and a lower abundance of *Agathobacter* at the genus level compared with grade 1/2 irAE patients (**Supplementary Figure S2F**).

Considering that there were differences between groups with respect to some demographic and clinical characteristics, we further compared microbiota differences in irAE patients according to gender, age, tumor type, and disease stage to exclude potential confounding effects of these clinical and pathological variables. PCoA revealed no significant differences in microbiota composition with respect to gender, age, tumor type, and disease stage (**Supplementary Figures S3A-D**).

### 3.3 Differences in the gut microbiota of patients with colitic and non-colitic irAEs

**Figure 3:**
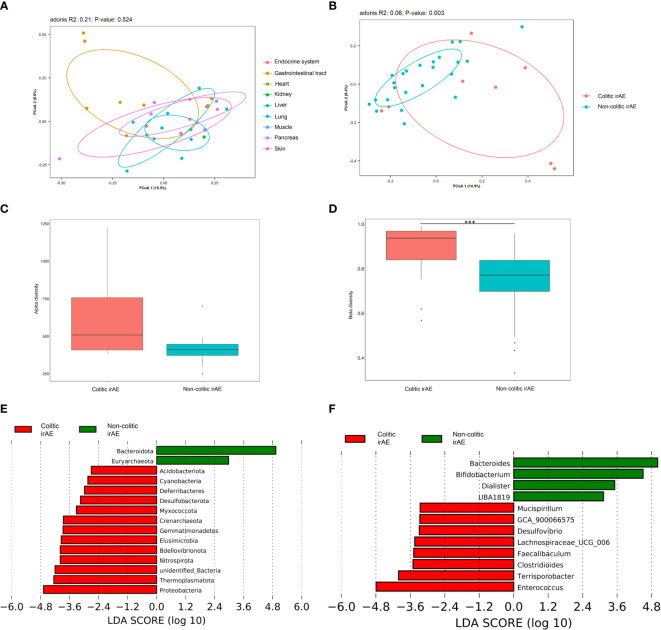
The gut microbiota of patients with colitic- and non-colitic-type irAEs. **(A)** PCoA plot of microbiota in patients with different kinds of irAEs; **(B)** PCoA plot of intestinal microbiota of patients with colitic and non-colitic irAEs; Box plots of α-diversity by observed species **(C)** and β-diversity by the Bray-Curtis method **(D)** of intestinal microbiota in patients with colitic and non-colitic irAEs; LEfSe analysis of intestinal microbiota at the phylum **(E)** and genus **(F)** levels in patients with colitic- and non-colitic irAEs. Abbreviations: irAE, immune-related adverse event; LEfSe, LDA effect size; PCoA, principal coordinates analysis. ***P < 0.001.

IrAEs involved a variety of different organs. There were 162 common OTUs and 1281, 83, 87, 13, 55, 12, 26, 25, and 50 differential OTUs in patients with irAEs involving the gastrointestinal tract, liver, lung, muscle, pancreas, skin, endocrine, heart, and kidney, respectively. The PCoA plot also revealed that the gut microbiota of patients with irAEs involving the gastrointestinal tract was quite distinct from patients with irAEs involving other organs (**Figure 3A**).

We speculated that occurrence of colitic type irAEs was different to the other types and especially related to the gut microbiota. Thus, irAEs were further classified into colitic and non-colitic irAEs. There were no significant differences in the demographic and clinical characteristics of these two groups, except for the tumor type (*P*=0.02) (**Supplementary Table 4**). There were 1159 common OTUs and 1555 and 588 differential OTUs in colitic and non-colitic irAE patients, respectively. The PCoA plot also revealed a significant difference in gut microbiota composition between these two groups (Adonis: *P*=0.003; MRPP: *P*=0.006; AMOVA: *P*=0.002) (**Figure 3B**). The median α-diversity in colitic irAE patients was higher than that of non-colitic irAE patients, with a non-significant trend in differences in observed species (*P*=0.10) (**Figure 3C**). The β-diversity was significantly different between groups by both Bray-Curtis and weighted Unifrac distances (*P*<0.001) (**Figure 3D**). LEfSe analysis of the main differences detected a lower abundance of *Bacteroidota* and a higher abundance of *Proteobacteria* at the phylum level and a lower abundance of *Bacteroides* and *Bifidobacterium* and higher abundance of *Enterococcus* at the genus level in colitic irAE compared with non-colitic irAE (**Figures 3E, F**).

### 3.4 The causal role of the gut microbiota in immune-related colitis development in a humanized microbiota mouse model

Although the above analysis revealed an association between the gut microbiota and irAEs, the causal relationship between the microbiota and irAEs or vice versa remained uncertain. Therefore, we performed FMT experiments using human donor feces in mice to explore causality.

**Figure 4:**
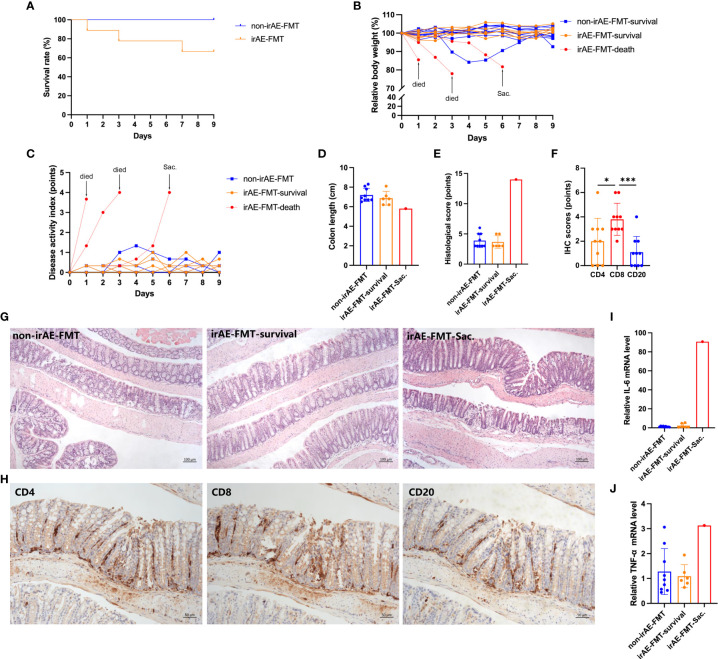
Comparison of colitis in colitic-irAE-FMT and non-irAE-FMT mice. **(A)** Survival curve; **(B)** Relative body weight change; **(C)** DAI scores; **(D)** Colon length; **(E)** Histological scoring of colonic tissue; **(F)** IHC scoring of CD4, CD8, and CD20 in colonic tissue from mice developing colitis; **(G)** HE-stained mouse colon tissue sections (100×); **(H)** CD4, CD8, and CD20 expression in mouse developing colitis by IHC (100×); **(I)** Colonic *Il6* mRNA levels; **(J)** Colonic *Tnf* mRNA levels by qRT-PCR with *Gapdh* as the internal reference. (Non-irAE-FMT n=9; irAE-FMT-survival n=6; irAE-FMT-death n=3; irAE-FMT-Sac. n=1). DAI, disease activity index; FMT, fecal microbiota transplantation; HE, hematoxylin-eosin; ICIs, immune checkpoint inhibitors; IHC, immunohistochemistry; *Il6*, interleukin-6; irAE, immune-related adverse event; *Tnf*, tumor necrosis factor. *P < 0.05, ***P < 0.001.

Three of nine colitic-irAE-FMT mice developed fatal severe colitis, while all nine non-irAE-FMT mice survived without colitis (*P*=0.052) (**Figure 4A**). Weight loss was more rapid and disease activity index (DAI) scores were higher in the three colitic mice than the other mice (**Figures 4B, C**). The irAE-FMT-Sac. mouse had a shorter colon (5.8 cm) than non-irAE-FMT mice (7.2 ± 0.7 cm) and colitic-irAE-FMT-survival mice (6.9 ± 0.7 cm) (**Figure 4D**), and the histological score in the irAE-FMT-Sac. mouse (14 points) was higher than those in the non-irAE-FMT mice (3.89 ± 1.17 points) and colitic-irAE-FMT-survival mice (3.67 ± 1.03 points) (**Figures 4E, G**). The mean IHC scores in the irAE-FMT-Sac. mouse from high to low were CD8 3.8 ± 1.3 points, CD4 2.0 ± 1.9 points, and CD20 1.1 ± 1.3 points (**Figures 4F, H**). Relative colonic *Il6* and *Tnf* mRNA levels were also higher in the irAE-FMT-Sac. mouse (*Il6*: 90.68 and *Tnf*: 3.13) than the non-irAE-FMT mice (*Il6*: 1.12 ± 0.53 and *Tnf*: 1.28 ± 0.92) and colitic-irAE-FMT-survival mice (*Il6*: 2.26 ± 2.24 and *Tnf*: 1.09 ± 0.46) (**Figures 4I, J**).

Microbiota analyses showed the microbiota composition of donors with colitic irAE and without irAE were different at phylum and genus levels (**Supplementary Figure S4A**). Furthermore, longitudinal microbiota analysis revealed significant changes from baseline to after antibiotic treatment, although the microbiota was stable from FMT to euthanasia at phylum and genus levels in both colitic-irAE-FMT and non-irAE-FMT mice (**Supplementary Figures S4B, C**). After FMT, the microbiota differed between irAE-FMT mice and non-irAE-FMT mice. However, in irAE-FMT mice, there was no significant difference in microbiota composition between mice developing colitis (irAE-FMT-Sac.) or those not developing colitis (irAE-FMT-survival) (**Supplementary Figure S4D**). In colitic-irAE-FMT mice, there was a decrease in beneficial genera and increase in harmful genera with immune-related colitis development (**Supplementary Figure S4E**); the mean relative abundance of beneficial genera was lower and harmful genera higher in the irAE-FMT-Sac. mouse compared with irAE-FMT-survival and non-irAE FMT mice (**Supplementary Table 5** and **Supplementary Figure S4F**).

### 3.5 IrAE patients express microbiomes representing different metabolism pathways

**Figure 5:**
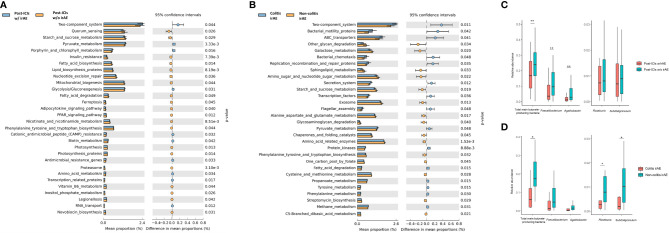
Functional predictions of the gut microbiota between different groups. Top 30 significantly different KEGG pathways with the highest abundance at level 3 between patients with and without irAEs **(A)** and between patients with colitic and non-colitic irAEs **(B)**; Box plot of main butyrate-producing bacteria abundance between patients with and without irAEs **(C)** and between patients with colitic- and non-colitic-type irAEs **(D)**. ICIs, immune checkpoint inhibitors; irAE, immune-related adverse event; KEGG, Kyoto Encyclopedia of Genes and Genome; w/, with; w/o, without. *P < 0.05, **P < 0.01.

We next performed functional prediction analysis to explore the underlying mechanisms by which the microbiota influence irAE development. The main differential pathways between patients who did and did not develop irAEs included the two-component system (*P*=0.044), starch and sucrose metabolism (*P*=0.029), pyruvate metabolism (*P*<0.001), mitochondrial biogenesis (*P*=0.044), and glycolysis/gluconeogenesis (*P*=0.031). Other differential pathways were lipid, amino acid, and vitamin metabolism including vitamin B6 metabolism (*P*=0.044), biotin metabolism (*P*=0.042), nicotinate and nicotinamide metabolism (*P*<0.001), amino acid metabolism (*P*=0.034), fatty acid biosynthesis (*P*=0.014), and fatty acid degradation (*P*=0.049) (**Figure 5A** and **Supplementary Figure S5**).

Differential pathways between patients with colitic and non-colitic irAEs included the two-component system (*P*=0.011), amino acid-related enzymes (*P*<0.001), ABC transporters (*P*=0.041), and exosomes (*P*=0.013). In addition to sugar and fatty acid pathways, the pathways of several amino acids were also significantly different including alanine, aspartate, and glutamate metabolism (*P*=0.017), cysteine and methionine metabolism (*P*=0.028), and phenylalanine, tyrosine, and tryptophan biosynthesis metabolism (*P*=0.032) (**Figure 5B** and **Supplementary Figure S6**).

As mentioned above, both *Faecalibacterium* and *Agathobacter* genera are butyrate-producing bacteria. Thus, we compared the abundance of these main butyrate-producing bacteria and found that the total abundance of butyrate-producing bacteria (*P*=0.007), *Faecalibacterium* (*P*=0.002), and *Agathobacter* (*P*=0.002) was lower in patients who developed irAEs (**Figure 5C**). There was also a lower abundance of the total main butyrate-producing bacteria (*P*=0.018), *Roseburia* (*P*=0.039), and *Subdoligranulum* (*P*=0.046) in colitic irAE compared with non-colitic irAE (**Figure 5D**).

### 3.6 Gut microbiota remained stable after ICI and irAE treatment

Finally, we assessed the relationship between microbiota and ICI and irAE treatment. Thirty-four pre-ICI samples from 34 patients and 50 post-ICI samples from 34 patients without irAEs were included in the analyses to compare the microbiota of patients before and after ICI therapy. There were 2395 common OTUs and 456 and 808 differential OTUs in pre- and post-ICI patients, respectively (**Supplementary Figure S7A**). Histograms of microbiota composition at phylum and genus levels and the PCoA plot revealed no significant differences between pre- and post-ICI patients (Adonis: *P*=0.96) (**Supplementary Figures S7B-D**). Also, the α-diversity by observed species index (*P*=0.66) and β-diversity by weighted Unifrac distance (*P*=0.19) were not significantly different (**Supplementary Figures S7E, F**).

We also compared the microbiota composition of irAE patients before and after irAE treatment (32 and 26 samples, respectively) and found no significant differences (Adonis: *P*=0.77). However, the β-diversity was higher by Bray-Curtis distance after irAE treatment (*P*=0.02) (**Supplementary Figure S8**).

## 4 Discussion

Here we explored the association between the gut microbiota and the development of irAEs. We show that: (i) the gut microbiota differs significantly in patients who do and do not develop irAEs after ICI treatment; (ii) the gut microbiota differs significantly between patients with colitic and non-colitic irAEs; (iii) the gut microbiota partially determined the occurrence of immune-related colitis in the mouse model; and (iv) patients with irAEs express microbiomes with different environmental information processing, genetic information processing, and metabolism pathways.

There have been several studies on the role of the gut microbiota in irAE occurrence. The baseline microbiota has been widely reported to shape irAE/immune-related colitis occurrence and development, but mainly in the context of metastatic melanoma and NSCLC by 16S sequencing or shotgun metagenomics (11, 12, 14, 15, 29, 30). Our study provides new data in different cancer types and adds further weight to a causal relationship between the microbiota and colitis.

There was no significant difference in α-diversity (species richness in the gut microbiome within a single sample) in patients who did and did not develop irAEs. However, the β-diversity, which measures microbial composition heterogeneity between samples, was higher in patients who developed irAEs compared with those who did not, consistent with previous studies (11, 15). We therefore speculate that the occurrence of irAEs depends more on microbiota composition (i.e., presence or absence of certain organisms) rather than the species richness. Indeed, patients who did not develop irAEs had a higher abundance of *Bifidobacterium*, *Faecalibacterium*, and *Agathobacter*. As a probiotic, *Bifidobacterium* protects against immune-related colitis by modulating Treg cell metabolism through an IL-10/IL-10Rα self-stimulatory loop and reducing levels of inflammatory cytokines IL-6, CSF3, and KC (31, 32). *Bifidobacterium* also appears to influence the clinical efficacy of ICIs (10), perhaps by modulating the activation of dendritic cells (33), and similarly *Faecalibacterium* also favors ICI efficacy (5, 12). Here we found that organisms in the *Faecalibacterium* genus tended to be more enriched in patients without irAEs, consistent with another study of patients receiving anti-PD-1 therapy (15). Conversely, Chaput et al. reported that abundant baseline *Faecalibacterium* was associated with colitis in patients receiving anti-CTLA-4 therapy (12). This discrepancy might be due to the context-dependent pro-inflammatory and anti-inflammatory effects of *Faecalibacterium* (34). *Agathobacter*, a butyrate-producing bacterium reported to be associated with ICI efficacy (13, 35), protected against irAEs in our study. We also compared the microbiota of patients with irAEs of different severity. Compared with those in mild-moderate irAE patients, the microbiota of severe irAE patients were more heterogeneous. Our results implicate the *Agathobacter* genus as protective against irAE occurrence and severity.

The microbiota of patients with colitic irAE was particularly different to those with irAEs affecting other organs. Patients with colitic irAE had a lower abundance of *Bacteroides* and *Bifidobacterium* genera. Usyk et al. recently reported that the presence of *Bacteroides vulgatus* and *Bacteroides dorei* predicts irAEs in patients with metastatic melanoma (14), and several studies have shown that higher representation of members of the *Bacteroidetes* phylum or its prominent genus *Bacteroides* is associated with protection from colitis (11, 12). This protective function might be related to the production of polysaccharide A, which might facilitate the development and function of Tregs (36). However, Andrews et al. found that *Bacteroides intestinalis* upregulated mucosal IL-1β and mediated immune-related colitis (37), suggesting that a more detailed species-level analysis is warranted.

It is worth considering whether microbiota differences resulted in immune-related colitis or whether the immune-related colitis disturbed the gut microbiota. This is, of course, impossible to establish in an observational study of clinical data. However, preclinical studies have provided some clues. For example, pre-treatment with vancomycin worsened immune-related colitis in mice, while administration of *Bifidobacterium* or *Lactobacillus reuteri* both alleviated the colitis (31, 38). These data suggest that the microbiota composition contributes to the development of immune-related colitis and that supplementation with probiotics to alter the gut microbiota effectively alleviates colitis. Even when colitic irAE patients in whom the microbiota may be influenced by the irAE were excluded, there were still significant differences between patients with and without irAEs. This evidence favors microbiota contributing to the occurrence of irAEs. Furthermore, our experiments in mice indicated that the gut microbiome caused immune-related colitis. After antibiotic treatment and establishment of humanized gut microbiota from colitic-irAE patients and non-irAE patients, colitis only occurred in the colitic-irAE-FMT mice after combined anti-PD-1 and anti-CTLA-4 treatment. The higher representation of CD8 compared with CD4 and CD20 cells by IHC further indicated that ICI-related colitis occurred in mice. Mouse microbiota analysis also supported the findings of microbiota differences in patients with and without irAEs. However, perhaps not surprisingly, not all colitic-irAE-FMT mice developed colitis, since other immune factors are likely to be involved in the pathogenesis of microbiota-related colitis.

Our functional analyses of patients with and without irAEs revealed a set of differentially expressed pathways mainly involved in environmental information processing, genetic information processing, and metabolism, e.g., sugar, lipid, amino acid, and purine and vitamin metabolism. Further analysis revealed that these differences were particularly significant in colitic irAE, suggesting that the pathogenesis of immune-related colitis may be highly related to gut microbiota-mediated mechanisms. While Mager et al. found that the microbiome could modulate ICI efficacy through the actions of the bacteria-derived metabolite inosine (39), there are still very few studies on the mechanism of action of microbiota and irAEs. Microbiota-derived metabolites such as short-chain fatty acids (SCFAs), bacterial tryptophan catabolites, branched chain amino acids (BCAAs), and vitamins play a role in immune-mediated inflammatory diseases including autoimmune disease and IBD (40), providing clues about the role of metabolism in irAEs and immune-related colitis. Butyrate is one of the most important SCFAs and is widely reported to be associated with anti-tumor efficacy (41) and immune-related diseases, and Chen et al. reported that butyrate can alleviate anti-PD-1/PD-L1-related cardiotoxicity (42). We also found decreased abundance of butyrate-producing bacteria in patients with irAEs, especially colitic irAE. With respect to other metabolites, amino acids can undergo complex processing to produce toxic compounds such as amines, phenols/indoles, and sulphurous compounds (43). Furthermore, pyruvate has also been reported to enhance immune responses by inducing dendrite protrusion from intestinal CX3CR1^+^ cells (44). We also observed differences in B vitamin pathways (vitamin B6, biotin, and niacin) in irAE patients, which are reported to regulate host immunity (45, 46).

There is still relatively little information on genetic information processing pathways in irAE patients, although several studies on breast cancer and IBD have reported similar findings (47, 48). The two-component system pathway was also more abundant in irAE patients, especially those with colitic irAE, perhaps reflecting bacterial responses to external stimuli. We speculate that these pathways might mediate irAEs by affecting bacterial growth, replication, and signaling, thereby affecting bacterial functions such as metabolism.

We also performed random forest modelling to distinguish patients with and without irAEs, and the resultant model achieved good performance for predicting irAEs. Of the three leading genera in the model, *Atopobium parvulum* is a key network hub of H_2_S producers that induces colitis (49), and it has previously been implicated in the development of intramucosal carcinomas (50). *Gemella* (51) and *Dubosiella* (52), involved in the synthesis of short-chain fatty acids (SCFAs), may also be related to protection from colitis. However, as random forests are nonlinear, it is difficult to explain the functional contribution of the individual biomarkers based on previous studies or putative mechanisms. Although there was no obvious change in the gut microbiota before and after ICI treatment, whether a predictive model based on post-ICI microbiota is the best choice of sample for predictive biomarkers still needs to be verified. Furthermore, the small sample size might lead to model overfitting, so extensive external validation is also required.

Interestingly, we found that the gut microbiota did not significantly change after anti-PD-1 therapy or irAE treatment, further suggesting that the baseline microbiota may shape ICI efficacy and irAE occurrence, and the microbiota remained stable after therapy initiation (12, 14, 30). Two recent clinical studies showed that FMT from ICI responders to primary non-responders partially reversed ICI efficacy in melanoma patients (8, 9). All these data suggest that altering the baseline microbiota may contribute to improving the ICI efficacy and avoiding irAEs.

There are several limitations to our study. The sample size was relatively small and the incidence of irAEs generally low (53). Therefore, subgroup analyses may have lacked statistical power or were not possible, for instance for comparing the microbiota of patients with different types of irAE. Furthermore, the efficacy of anti-PD-1 therapy combined with chemotherapy can be as high as 84.6% in advanced NSCLC patients (54), so we could not analyze associations between microbiota composition and ICI efficacy. We prospectively collected fecal samples from patients initially treated with ICIs, and only 3/37 patients developed irAEs, so baseline fecal samples before ICI treatment were generally lacking. Finally, functional analyses were performed based on 16S rDNA amplicon sequencing rather than the newer, more granular metagenomic sequencing and metabolomics analyses.

In conclusion, we detected significant differences in the gut microbiota of patients with and without irAEs and between patients with colitic and non-colitic irAE. FMT experiments from humans to mice indicated a causal link between the gut microbiota and immune-related colitis. We also built a predictive irAE model to identify patients at high risk of irAEs, who may particularly benefit from strategies to prevent irAEs such as by altering the gut microbiota. IrAEs, especially immune-related colitis, seem to be driven by metabolic mechanisms. The roles of the gut microbiota in irAEs and immune-related colitis require further verification in larger studies, and the predictive model for irAEs needs validating in external, independent patient cohorts. Metabolomics research is now needed to better identify and understand the protective protagonists and potential underlying protective mechanisms. Supplementation with probiotics to prevent and attenuate irAEs should also be considered given that they are generally safe and easy to administer.

## Data availability statement

The data that support the findings of this study are openly available in figshare at http://doi.org/10.6084/m9.figshare.21431871.

## Ethics statement

The studies involving human participants were reviewed and approved by Ethical Committee of Peking Union Medical College Hospital (No. ZS-3037). The patients/participants provided their written informed consent to participate in this study. The animal study was reviewed and approved by Experimental Animal Ethics Committee of Peking Union Medical College Hospital (XHDW-2022-066).

## Author contributions

Analyzed and interpreted data, drafted the manuscript: XL. Animal experiments and analyzed data: HT, QZ. Analyzed microbiota data: YZ, DC, YL. Enrolled patients and collected samples: MC, JZ, YX. Supervised and supported the study: MW, JQ. Designed and performed the study, critically revised the manuscript, funding support: BT. The work reported in the paper was performed by the authors.
