# Supplementary material for: Gut microbiota composition in patients with advanced malignancies experiencing immune-related adverse events
Source: Front Immunol. 2023 Feb 20;14:1109281. doi: 10.3389/fimmu.2023.1109281 (PMC9986626; doi:10.3389/fimmu.2023.1109281)
Supplement: Supplementary file 1 [file DataSheet_1.docx]

Supplementary Material

Gut Microbiota Composition in Patients with Advanced Malignancies Experiencing Immune-related Adverse Events

**Supplementary Table 1 Primer sequence information**

| Species | Gene | F/R | Primer sequence (5'—3') |
| --- | --- | --- | --- |
| Mouse | *Il6* | Forward | ACCAGAGGAAATTTTCAATAGGC |
| Mouse | *Il6* | Reverse | TGATGCACTTGCAGAAAACA |
| Mouse | *Tnf* | Forward | AGGGTCTGGGCCATAGAACT |
| Mouse | *Tnf* | Reverse | CCACCACGCTCTTCTGTCTAC |

**Supplementary Table 2 Demographic and characteristics of patients with and without irAEs**

| Characteristic | | Patients with irAEs  (N=32) | | Patients without irAEs  (N=34) | | *P* value | |
| --- | --- | --- | --- | --- | --- | --- | --- |
| Age - Median (IQR) - years | | 64.5 (54.75-68.25) | | 64 (61.25-69.75) | | 0.240 | |
| Male sex – no. (%) | | 26 (81.3%) | | 30 (88.2%) | | 0.505 | |
| Tumor type – no. (%) | | | | | | | |
| Lung adenocarcinoma | | 12 (37.5%) | | 16 (47.1%) | | 0.226 | |
| Squamous cell lung cancer | | 9 (28.1%) | | 14 (41.2%) | |  |  |
| Small cell lung cancer | | 3 (9.4%) | | 1 (2.9%) | |  |  |
| Other types of lung cancer | | 1 (3.1%) | | 1 (2.9%) | |  |  |
| Tumors of other organs | | 7 (21.9%) | | 2 (5.9%) | |  |  |
| Disease stage – no. (%) | | | | | | | |
| Stage II | | 0 (0.0%) | | 1 (2.9%) | | 0.051 | |
| Stage III | | 7 (21.9%) | | 15 (44.1%) | |  |  |
| Stage IV | | 25 (78.1%) | | 18 (52.9%) | |  |  |
| Combined chemotherapy or targeted therapy – no. (%) | | 29 (90.6%) | | 33 (97.1%) | | 0.348 | |
| Progression-free survival - Median (IQR) - days | | 249.5 (158.75-338.75) | | 160 (111.5-236.25) | | 0.027 | |
| ICIs efficacy– no. (%) | | | | | | | |
| Partial response (PR) | | 18 (56.3%) | | 18 (52.9%) | | 1.000 | |
| Stable disease (SD) | | 13 (40.6%) | | 14 (41.2%) | |  |  |
| Progressive disease (PD) | | 1 (3.1%) | | 2 (5.9%) | |  |  |
| Therapy lines– no. (%) | | | | | | | |
| 1^st^ line | | 25 (78.1%) | | 27 (78.4%) | | 0.902 | |
| 2^nd^ line | | 5 (15.6%) | | 6 (18.9%) | |  |  |
| >3^rd^ line | | 2 (6.3%) | | 1 (2.7%) | |  |  |
| irAE grade– no. (%) | | | | | | | |
| Grade 1-2 | | 13 (40.6%) | | — | | — | |
| Grade 3-4 | | 19 (59.4%) | | — | |  |  |
| Involved organs of irAE– no. (%) | | | | | | | |
| Mono | | 27 (84.4%) | | — | | — | |
| Multiple | | 5 (15.6%) | | — | |  |  |
| Involved organ of irAE (cases) | | | | | | | |
| Gastrointestinal tract | | 8 (21.1%) | | — | | — | |
| Lung | | 7 (18.4%) | | — | |  |  |
| Pancreas | | 6 (15.8%) | | — | |  |  |
| Liver | | 5 (13.2%) | | — | |  |  |
| Skin | | 4 (10.5%) | | — | |  |  |
| Heart | | 3 (7.9%) | | — | |  |  |
| Kidney | | 3 (7.9%) | | — | |  |  |
| Muscle | | 1 (2.6%) | | — | |  |  |
| Endocrine | | 1 (2.6%) | | — | |  |  |

ICIs, immune checkpoint inhibitors; irAE, immune-related adverse events; IQR, interquartile range; PR, partial response; PD, progressive disease; SD, stable disease

**Supplementary Table 3 Demographic and characteristics of patients with different severities of irAEs**

| Characteristic | Patients with grade 1-2 irAEs (N=13) | Patients with grade 3-4 irAEs (N=19) | *P* value |
| --- | --- | --- | --- |
| Age - Median (IQR) years | 63 (58-69) | 66 (53.5-68) | 0.734 |
| Male sex – no. (%) | 8 (61.5%) | 18 (94.7%) | 0.029 |
| Tumor type – no. (%) | | | |
| Lung adenocarcinoma | 7 (53.8%) | 5 (26.3%) | 0.259 |
| Squamous cell lung cancer | 3 (23.1%) | 6 (31.6%) |  |
| Small cell lung cancer | 0 (0.0%) | 3 (15.8%) |  |
| Other types of lung cancer | 1 (7.7%) | 0 (0.0%) |  |
| Tumors of other organs | 2 (15.4%) | 5 (26.3%) |  |
| Disease stage – no. (%) | | | |
| Stage III | 2 (15.4%) | 5 (26.3%) | 0.389 |
| Stage IV | 11 (84.6%) | 14 (73.7%) |  |
| Combined chemotherapy or targeted therapy – no. (%) | 13 (100.0%) | 16 (84.2%) | 0.253 |
| Progression-free survival - Median (IQR) - days | 243 (178-290) | 262 (131.5-396) | 0.791 |
| ICIs efficacy– no. (%) | | | |
| Partial response (PR) | 7 (53.8%) | 11 (57.9%) | 0.838 |
| Stable disease (SD) | 6 (46.2%) | 7 (36.8%) |  |
| Progressive disease (PD) | 0 (0.0%) | 1 (5.3%) |  |
| Therapy lines– no. (%) | | | |
| 1^st^ line | 10 (76.9%) | 15 (78.9%) | 0.210 |
| 2^nd^ line | 1 (7.7%) | 4 (21.1%) |  |
| >3^rd^ line | 2 (15.4%) | 0 (0.0%) |  |

ICIs, immune checkpoint inhibitors; irAE, immune-related adverse events; IQR, interquartile range; PR, partial response; PD, progressive disease; SD, stable disease

**Supplementary Table 4 Demographic and characteristics of patients with different types of irAEs**

| Characteristic | Patients with colitic irAEs  (N=8) | Patients with non-colitic irAEs (N=24) | *P* value |
| --- | --- | --- | --- |
| Age - Median (IQR) - years | 67 (63.75-69.25) | 63 (53.75-67.25) | 0.174 |
| Male sex – no. (%) | 8 (100.0%) | 18 (75.0%) | 0.149 |
| Tumor type – no. (%) | | | |
| Lung adenocarcinoma | 1 (12.5%) | 11 (45.8%) | 0.020 |
| Squamous cell lung cancer | 6 (75.0%) | 3 (12.5%) |  |
| Small cell lung cancer | 0 (0.0%) | 3 (12.5%) |  |
| Other types of lung cancer | 0 (0.0%) | 1 (4.2%) |  |
| Tumors of other organs | 1 (12.5%) | 6 (25.0%) |  |
| Disease stage – no. (%) | | | |
| Stage III | 2 (25.0%) | 5 (20.8%) | 1.000 |
| Stage IV | 6 (75.0%) | 19 (79.2%) |  |
| Combined chemotherapy or targeted therapy – no. (%) | 6 (75.0%) | 23 (95.8%) | 0.147 |
| Progression-free survival - Median (IQR) - days | 216 (156-293.75) | 249.5 (159.5-354.25) | 0.848 |
| ICIs efficacy– no. (%) | | | |
| Partial response (PR) | 7 (87.5%) | 11 (45.8%) | 0.119 |
| Stable disease (SD) | 1 (12.5%) | 12 (50.0%) |  |
| Progressive disease (PD) | 0 (0.0%) | 1 (4.2%) |  |
| Therapy lines– no. (%) | | | |
| 1^st^ line | 8 (100.0%) | 17 (70.8%) | 0.435 |
| 2^nd^ line | 0 (0.0%) | 5 (20.8%) |  |
| >3^rd^ line | 0 (0.0%) | 2 (8.3%) |  |

ICIs, immune checkpoint inhibitors; irAE, immune-related adverse events; IQR, interquartile range; PR, partial response; PD, progressive disease; SD, stable disease

**Supplementary Table 5 Relative abundance of key genera in mice microbiota**

| Genus | Group | Timepoint | Relative abundance |
| --- | --- | --- | --- |
| *Bifidobacterium* | Colitic-irAE-FMT mice developing colitis | Before antibiotics treatment | 0.00004 ± 0.00007 |
|  |  | After antibiotics treatment | 0.00002 ± 0.00003 |
|  |  | After FMT | 0 ± 0 |
|  |  | Before euthanasia | 0 |
|  | Colitic-irAE-FMT mice without colitis | Before antibiotics treatment | 0 ± 0 |
|  |  | After antibiotics treatment | 0.00001 ± 0.00002 |
|  |  | After FMT | 0 ± 0 |
|  |  | Before euthanasia | 0.00004 ± 0.00008 |
|  | Non-irAE-FMT | Before antibiotics treatment | 0.00005 ± 0.00005 |
|  |  | After antibiotics treatment | 0 ± 0 |
|  |  | After FMT | 0 ± 0 |
|  |  | Before euthanasia | 0.00002 ± 0.00003 |
| *Faecalibacterium* | Colitic-irAE-FMT mice developing colitis | Before antibiotics treatment | 0.00071 ± 0.00116 |
|  |  | After antibiotics treatment | 0.00055 ± 0.00060 |
|  |  | After FMT | 0 ± 0 |
|  |  | Before euthanasia | 0.00005 |
|  | Colitic-irAE-FMT mice without colitis | Before antibiotics treatment | 0.00021 ± 0.00036 |
|  |  | After antibiotics treatment | 0.00396 ± 0.00545 |
|  |  | After FMT | 0 ± 0 |
|  |  | Before euthanasia | 0.00057 ± 0.00091 |
|  | Non-irAE-FMT | Before antibiotics treatment | 0.00353 ± 0.00453 |
|  |  | After antibiotics treatment | 0.00412 ± 0.00233 |
|  |  | After FMT | 0.00274 ± 0.00044 |
|  |  | Before euthanasia | 0.00511 ± 0.00246 |
| *Agathobacter* | Colitic-irAE-FMT mice developing colitis | Before antibiotics treatment | 0.00018 ± 0.00031 |
|  |  | After antibiotics treatment | 0.00028 ± 0.00031 |
|  |  | After FMT | 0 ± 0 |
|  |  | Before euthanasia | 0 |
|  | Colitic-irAE-FMT mice without colitis | Before antibiotics treatment | 0.00008 ± 0.00014 |
|  |  | After antibiotics treatment | 0.00070 ± 0.00065 |
|  |  | After FMT | 0 ± 0 |
|  |  | Before euthanasia | 0.00032 ± 0.00056 |
|  | Non-irAE-FMT | Before antibiotics treatment | 0.00036 ± 0.00039 |
|  |  | After antibiotics treatment | 0.00028 ± 0.00020 |
|  |  | After FMT | 0.00031 ± 0.00007 |
|  |  | Before euthanasia | 0.00061 ± 0.00026 |
| *Erysipelatoclostridium* | Colitic-irAE-FMT mice developing colitis | Before antibiotics treatment | 0.00334 ± 0.00250 |
|  |  | After antibiotics treatment | 0.00249 ± 0.00267 |
|  |  | After FMT | 0.00737 ± 0.00178 |
|  |  | Before euthanasia | 0.00302 |
|  | Colitic-irAE-FMT mice without colitis | Before antibiotics treatment | 0.00188 ± 0.00092 |
|  |  | After antibiotics treatment | 0.00047 ± 0.00049 |
|  |  | After FMT | 0.00439 ± 0.00163 |
|  |  | Before euthanasia | 0.00756 ± 0.00502 |
|  | Non-irAE-FMT | Before antibiotics treatment | 0.00063 ± 0.00045 |
|  |  | After antibiotics treatment | 0 ± 0 |
|  |  | After FMT | 0.00369 ± 0.00069 |
|  |  | Before euthanasia | 0.00159 ± 0.00024 |

FMT, faecal microbiota transplantation; irAE, immune-related adverse events.

**
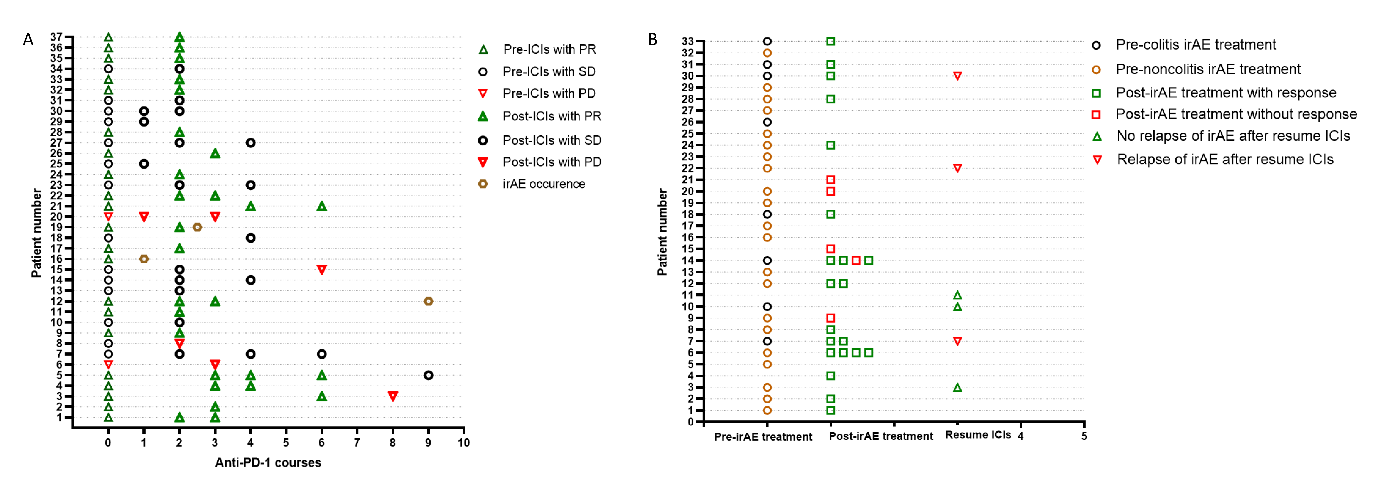
**

**Figure S1.** **Fecal sample collection in anti-PD-1 therapy patients and irAE patients**

Abbreviations: PD-1, programmed death-1; PR, partial response; SD, stable disease; PD, progressive disease; irAE, immune-related adverse event; ICIs, immune checkpoint inhibitors.


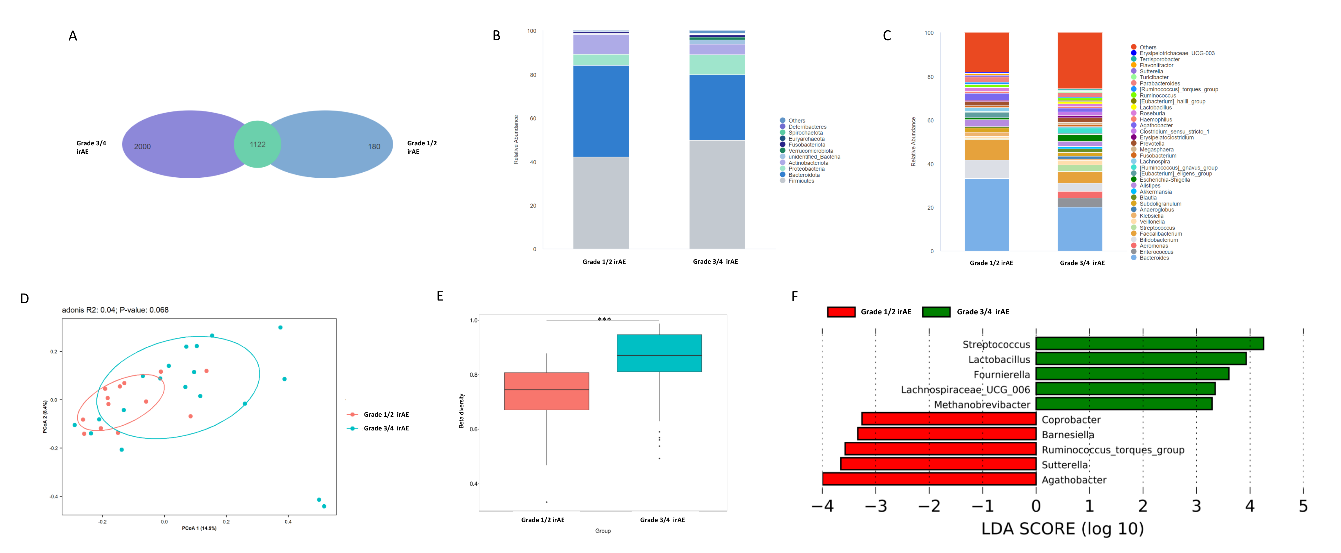


**Figure S2. The gut microbiota of patients with different irAE severities**

(A) Venn diagram of OTUs; Histogram of intestinal microbiota at the phylum (B) and genus (C) levels; (D) PCoA plot; (E) Box plot of β-diversity by Bray-Curtis distance; (F) LEfSe analysis of intestinal microbiota at the genus level between patients with grade 1/2 irAEs and grade 3/4 irAEs.

Abbreviations: ICIs, immune-checkpoint inhibitors; irAE, immune-related adverse event; LEfSe, LDA effect size; OTU, operational taxonomic unit; PCoA, principal coordinates analysis. **P* < 0.05, ***P* < 0.01, ****P* < 0.001.


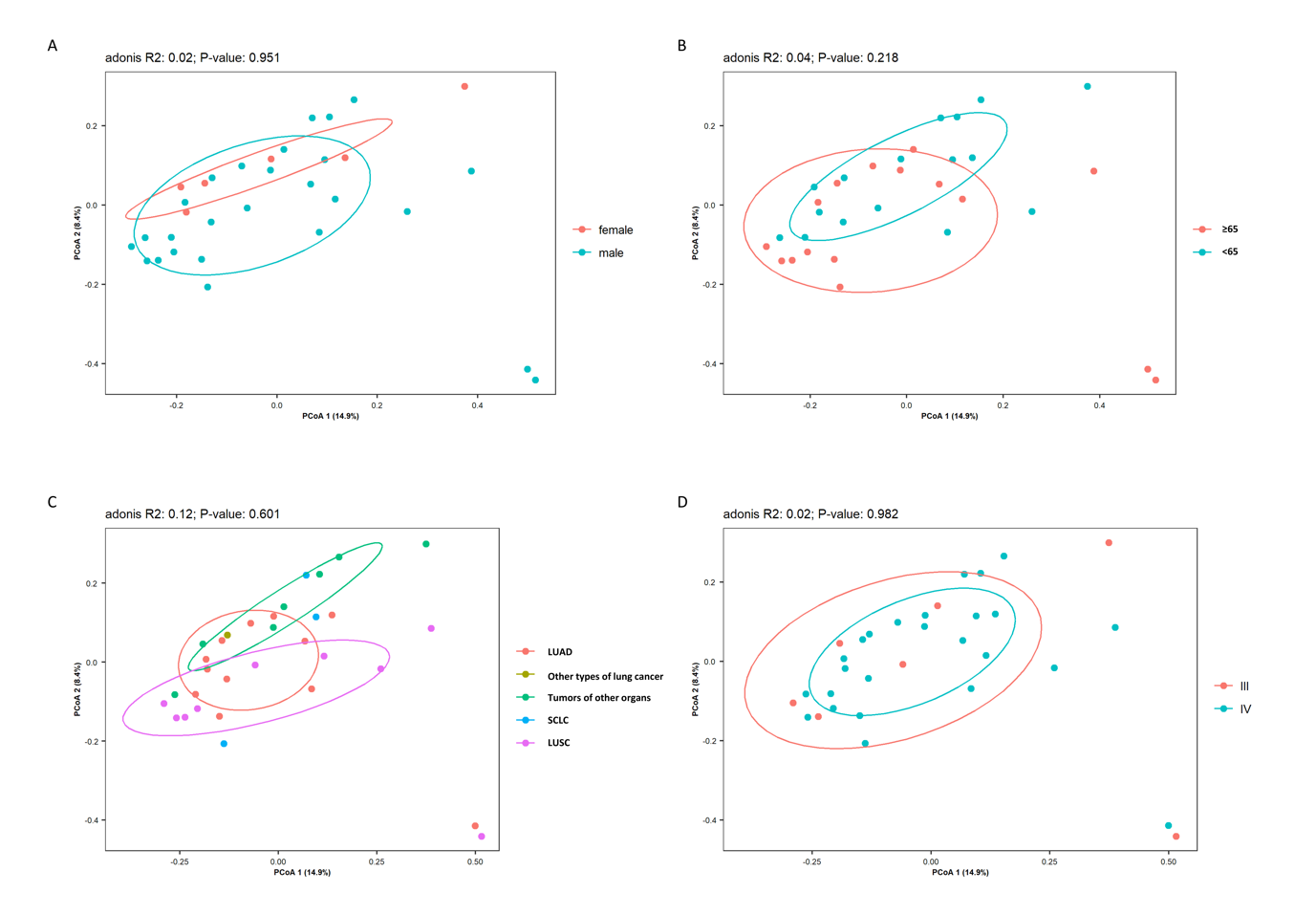


**Figure S3. The intestinal microbiota of patients with different demographic and clinical characteristics**

PCoA plots of the intestinal microbiota of patients within different (A) gender, (B) age, (C) tumor type, and (D) disease stage.

Abbreviations: PCoA, principal coordinates analysis.


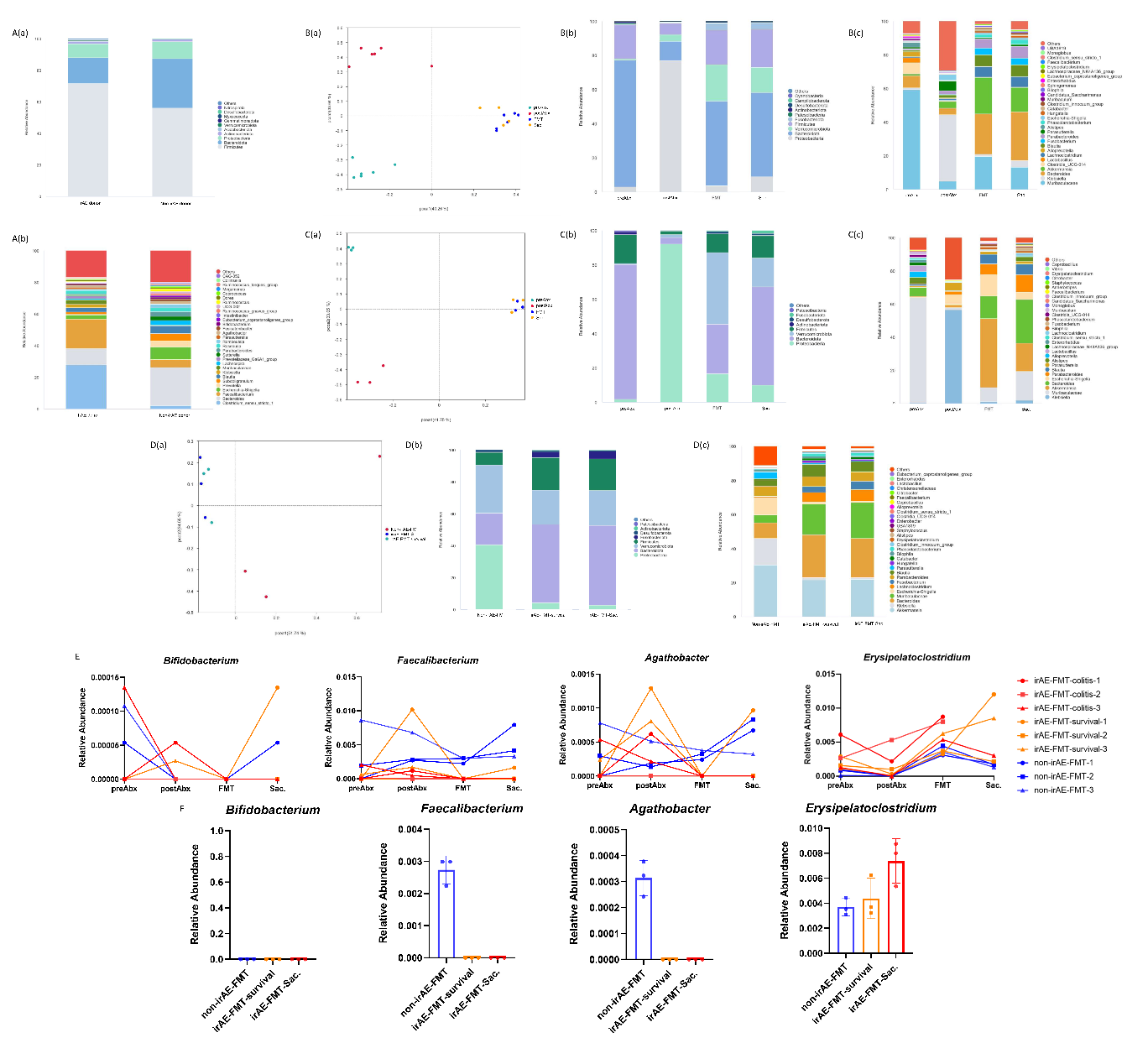


**Figure S4. The intestinal microbiota of FMT donors and mice**

(A) Histograms of microbiota composition of donors for colitic-irAE-FMT and non-irAE-FMT mice at the phylum and genus levels; PCoA plots and histograms at the phylum and genus levels of microbiota composition of colitic-irAE-FMT (B) and non-irAE-FMT mice (C) before and after antibiotics, after FMT, and before euthanasia; (D) PCoA plots and histograms at phylum and genus levels of microbiota after FMT of non-irAE-FMT mice, irAE-FMT mice developing colitis, and irAE-FMT mice without colitis; (E) Relative abundance of several genera of mice microbiota at the points of before antibiotic treatment, after antibiotic treatment, after FMT treatment, and before euthanasia; (F) Relative abundance of several genera of mice microbiota after FMT of non-irAE-FMT mice, irAE-FMT mice developing colitis, and irAE-FMT mice without colitis

Abbreviations: FMT, faecal microbiota transplantation; irAE, immune-related adverse event; PCoA, principal coordinates analysis.


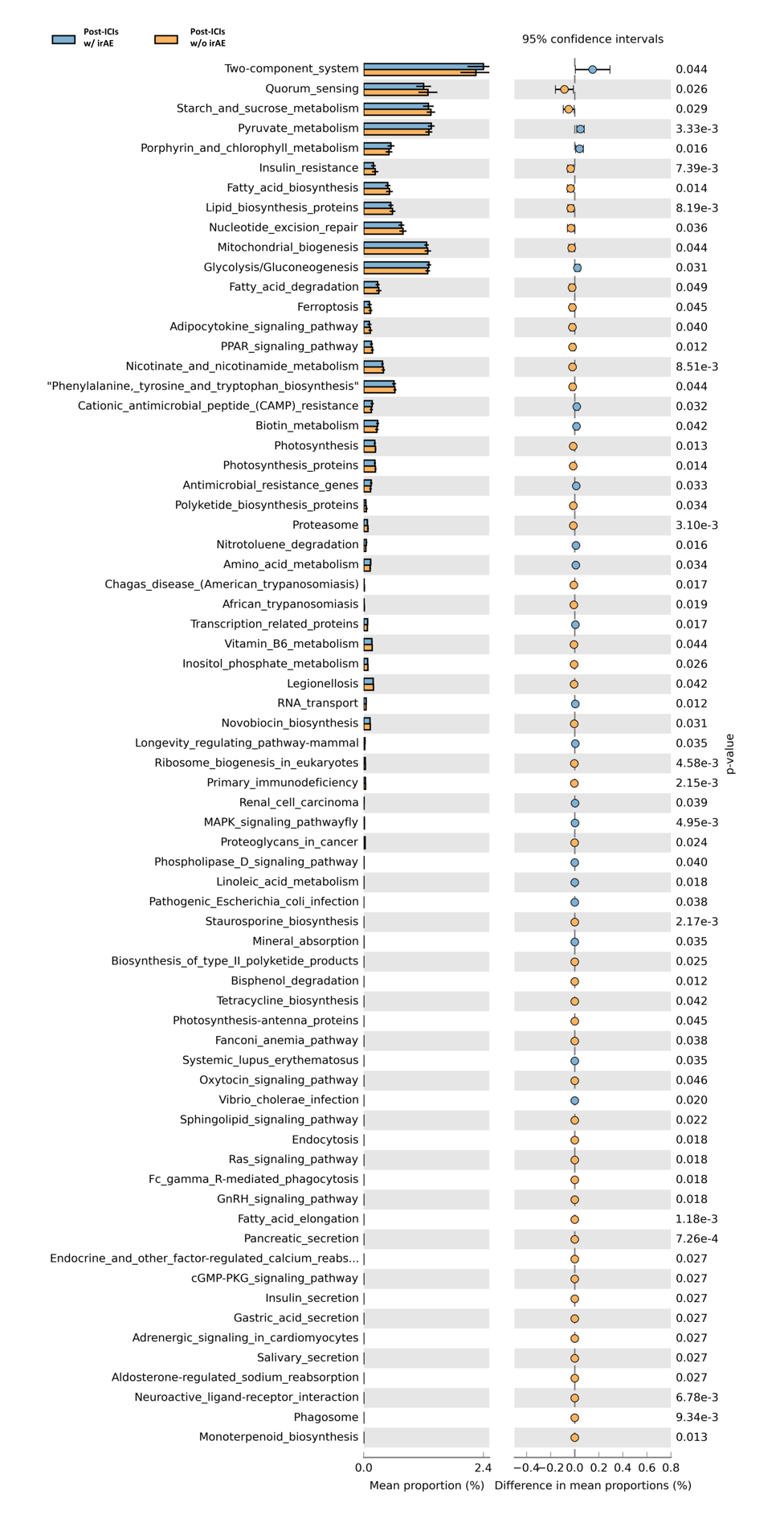


**Figure S5. Significantly different KEGG pathways between patients developing and not developing irAEs**

Abbreviations: irAE, immune-related adverse event; KEGG, Kyoto Encyclopedia of Genes and Genomes.

**
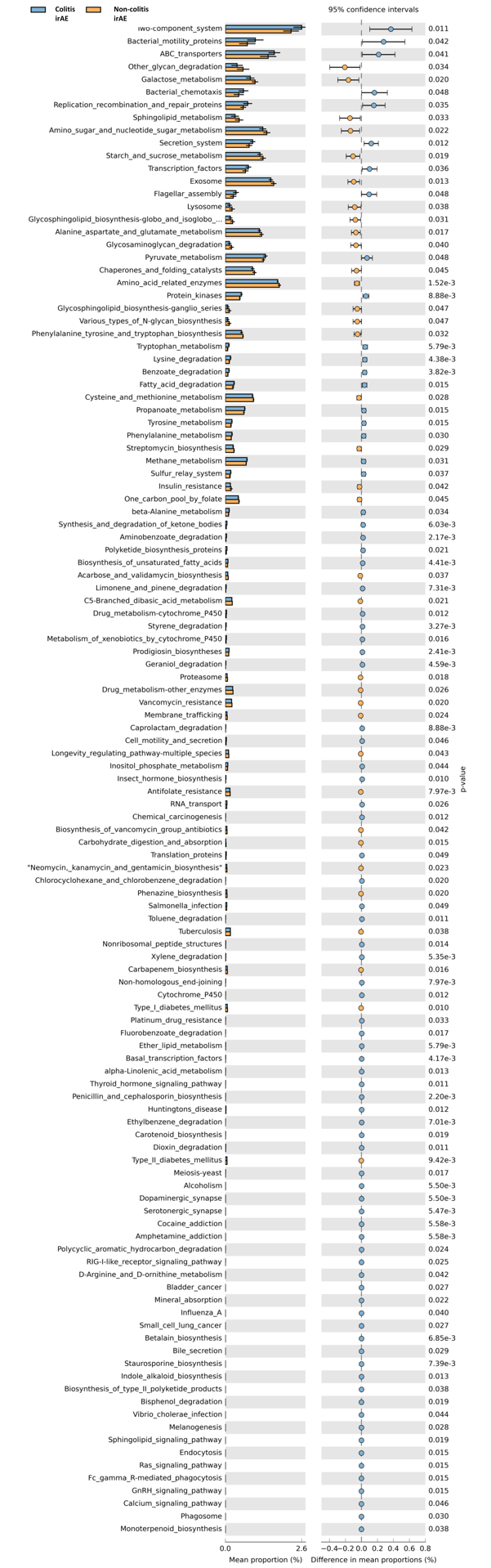
**

**Figure S6. Significantly different KEGG pathways between patients developing and not developing colitis-type irAEs**

Abbreviations: irAE, immune-related adverse event; KEGG, Kyoto Encyclopedia of Genes and Genomes.


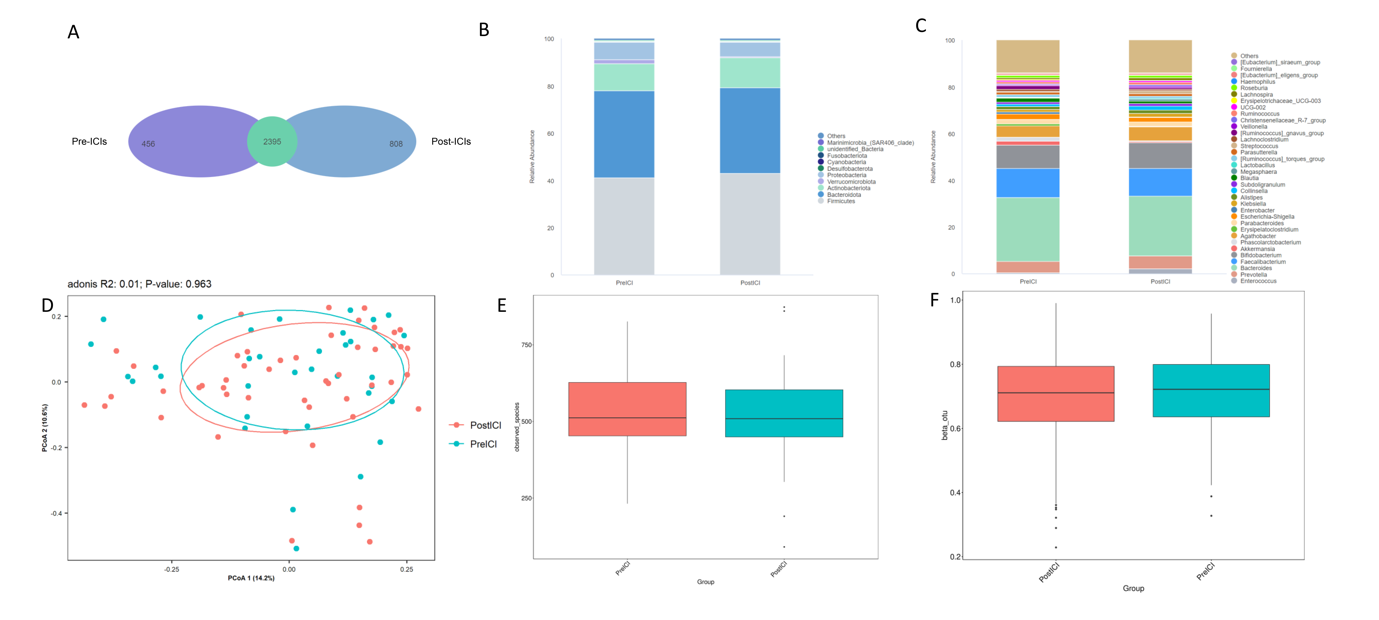


**Figure S7. The intestinal microbiota of patients pre-ICIs and post-ICIs**

(A) Venn diagram of OTUs; (B) Histogram of intestinal microbiota at the phylum level; (C) Histogram of intestinal microbiota at the genus level; (D) PCoA plot; (E) Box plot of α diversity by observed species; (F) Box plot of β diversity by Bray-Curtis distance.

Abbreviations: ICIs, immune-checkpoint inhibitors; OTUs, operational taxonomic units; PCoA, principal coordinates analysis.


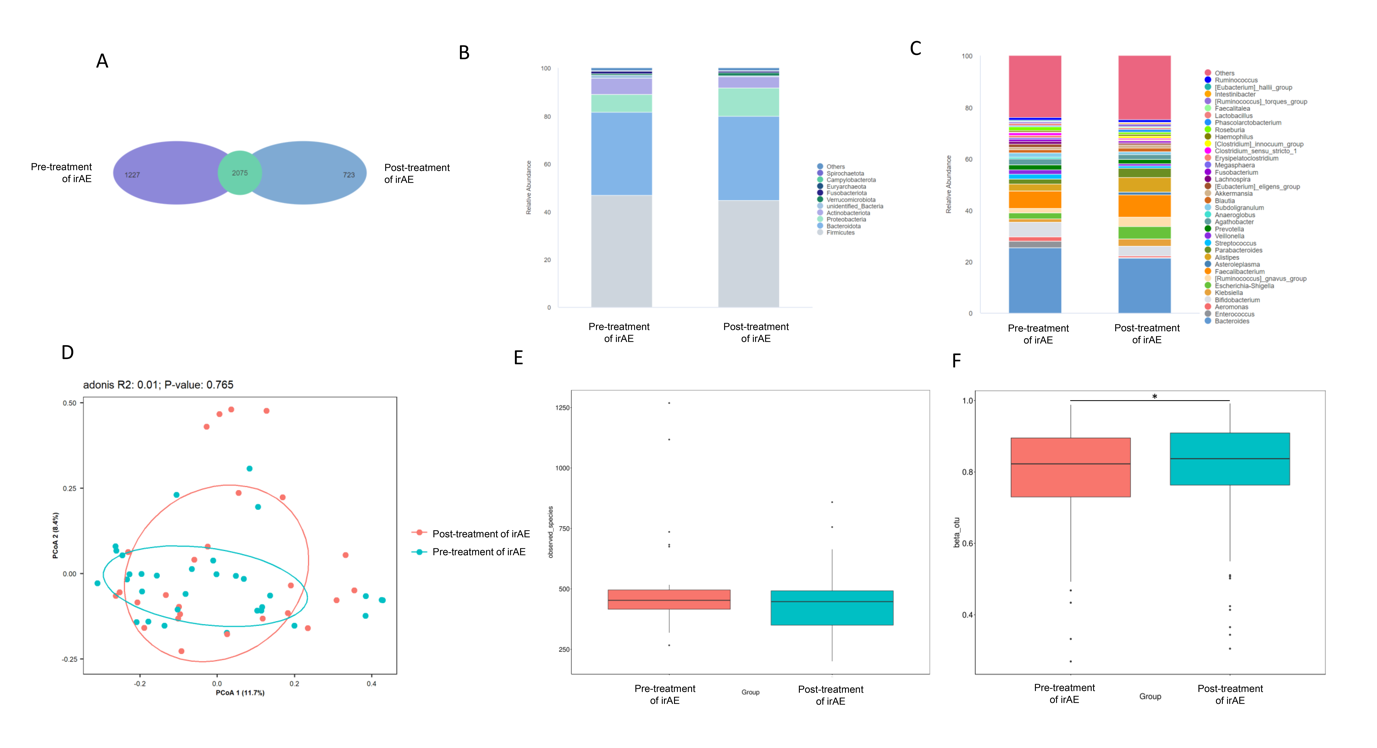


**Figure S8. The intestinal microbiota of patients pre and post irAE treatment**

(A) Venn diagram of OTUs; (B) Histogram of intestinal microbiota at the phylum level; (C) Histogram of intestinal microbiota at the genus level; (D) PCoA plot; (E) Box plot of α diversity by observed species; (F) Box plot of β diversity by weighted-Unifrac.

Abbreviations: irAE, immune-related adverse event; OTUs, operational taxonomic units; PCoA, principal coordinates analysis.
